# Improving usability of the STOPP/START version 3 criteria: development of a practice tool for clinicians, students and researchers

**DOI:** 10.1007/s41999-025-01214-y

**Published:** 2025-06-17

**Authors:** O. Dalleur, F. X. Sibille, A. Mouzon, F. Vaillant, S. Marien, A. Spinewine, B. Boland

**Affiliations:** 1 Clinical Pharmacy & Pharmacoepidemiology Research Group, Louvain Drug Research Institute, UCLouvain, Brussels, Belgium; 2 Groupe de Recherche en Gériatrie & Gérontologie, Institut de Recherche Santé et Société (IRSS), UCLouvain, Brussels, Belgium; 3 Pharmacy Department, Cliniques universitaires Saint-Luc, Brussels, Belgium; 4 Geriatric Medicine Department, CHU UCL Namur, Yvoir, Belgium; 5 Pharmacy Department, CHU UCL Namur, Yvoir, Belgium; 6 Geriatric Medicine, Cliniques universitaires Saint-Luc, Brussels, Belgium

**Keywords:** Potentially inappropriate medication list, Practice patterns, Medication review, Older people, STOPP/START criteria

## Abstract

**Background:**

The STOPP/START criteria are aimed to be used by clinicians to perform medication review to optimize pharmacotherapy in older adults. The STOPP/START.version 3 (SS.v3) published in *European Geriatric Medicine* in 2023 updated and significantly extended the previous version. SS.v3 is more open to criticism for its poor usability in daily clinical practice. The present study aimed at contributing to SS.v3 by highlighting the new content of its criteria and by providing clinicians, students and researchers with a user-friendly presentation to facilitate medication review.

**Methods:**

The SS.v3 criteria were reviewed by seven geriatricians and clinical pharmacists with expertise in pharmacotherapy in older adults. The 190 SS.v3 criteria were carefully compared to the 115 SS.v2 criteria in order to identify the new ones and those with clinically significant modifications. A single-entry table was developed for the medications (STOPP.v3) and the medical conditions (START.v3).

**Results:**

Among the 133 STOPP.v3 criteria, we identified 54 new (41%) and 25 modified (19%) ones. The 57 START.v3 criteria include 24 new (42%) and 17 modified (30%) ones. The single-entry table is a comprehensive presentation of SSv3 in 3 pages. It is systematically organized and upfront presents the medication for the STOPP criteria and the condition for the START criteria, enhancing its usability.

**Conclusion:**

This work highlights many new and modified SS.v3 criteria that clinicians, students and researchers should be aware of. The single-entry table for STOPP medications and START clinical conditions may facilitate medication review for older adults in daily clinical practice.

**Supplementary Information:**

The online version contains supplementary material available at 10.1007/s41999-025-01214-y.

## Key summary points

**Aim:**

The present study aimed at contributing to STOPP/START.v3 by highlighting the new content of the criteria and by providing clinicians, students and researchers with a user-friendly presentation to facilitate medication review.

**Findings:**

More than half of STOPP/START.v3 criteria are new or significantly modified showing important update of this version. To improve the usability of the list, a practice tool (i.e. a single-entry table) is presented.

**Message:**

The adoption in clinical practice of the extended list of STOPP/START.v3 criteria could be improved by this user-friendly presentation.

**Supplementary Information:**

The online version contains supplementary material available at 10.1007/s41999-025-01214-y.

## Introduction

The eagerly anticipated STOPP/START (Screening Tool of Older Persons’ Prescriptions/Screening Tool to Alert to Right Treatment) version 3 (SS.v3) was developed by European experts in geriatric pharmacotherapy and published in the August 2023 issue of *European Geriatric Medicine*. The article provides a list of 190 explicit criteria to detect potentially inappropriate medications (PIM) and potential prescribing omissions (PPO) in older adults [1].

Several teams from various countries have since commented on the revised version and have critically evaluated its strengths and weaknesses [2–7]. A significant concern pertains to its usability. Firstly, the SS.v3 does not distinguish between the new, the modified and the unchanged criteria, as pointed out by Canadian colleagues [2]. This lack of distinction complicates the transition for users familiar with the previous version, making it challenging to implement the changes introduced in the new version. Secondly, the expanded list of 190 criteria poses a considerable challenge when conducting a medication review for older adults [5, 6]. Lastly, while the system-based approach of the STOPP/START lists is logically sound and beneficial for research, it proves to be less user-friendly in daily clinical practice for the clinician (physician or pharmacist) facing an older adult using a medication belonging to different sections. To find out whether a medication is potentially inappropriate indeed requires examining many sections. This is particularly the case for several frequently prescribed medications in older adults such as benzodiazepines (D8-10, G4 and K1), non-steroidal anti-inflammatory drugs (NSAIDs; B17, B19, C10, E4, H1, H2, H3, H6, H7) and corticosteroids (B19, F5, H4-5, G2) [5]. Given that reliable software applications are not widely accessible to most clinicians and their operationalisation remains challenging [6, 8], navigating through an extensive list of criteria to determine the potential inappropriateness of a medication continues to be a daunting task for clinicians.

Furthermore, the applicability of the tool, which is the extent to which the users can implement a recommendation into practice, based on internal qualities such as clearly defined eligible patients [9, 10], is under scrutiny. Notably, a recent Swedish evaluation indicated a diminishing trend in the applicability of the STOPP/START across observational studies with each successive version [11].

For these reasons, a user-friendly presentation is necessary. Indeed, when developing clinical practice tools such as STOPP/START, the emphasis is frequently placed on the development and validation of content, while the optimization of the design is often overlooked. It’s important to note that design characteristics, such as usability, play a crucial role in successful implementation and can also affect the likelihood of non-adoption [12]. The latest American Geriatrics Society Beers criteria® highlight their consideration for the formatting of the recommendation to enhance usability [13]. User-centered design stands as one of the most recognized strategies that can enhance the usability of clinical tools [12].

As geriatricians and clinical pharmacists using STOPP/START criteria since a decade in daily clinical care, academic teaching, and clinical research [8, 14–22] and more recently in the OPERAM trial [23], we intend to contribute to the usability of SSv3 by.

- Highlighting modifications and additions made in the SS.v3 as compared to version two (v2), and.
- Developing a more user-friendly presentation to facilitate medication review in clinical practice and teaching. We had already conducted this work for the French version 2 [24], which is widely utilized by a substantial number of clinicians and students in Belgium. Given the significant expansion in the number of criteria, we believed there was an opportunity to carry out similar work and make it available to a larger, English-speaking audience.

## Methods

This study is a contribution to the SS.v3 criteria by seven clinicians (three geriatricians and four hospital clinical pharmacists) experts in pharmacotherapy at the Université catholique de Louvain (UCLouvain), Belgium.

### Identification of the new and the modified criteria

First, two senior investigators (BB and OD) compared the lists of SS.v3 criteria [1] between the supplementary file 1 (the 190 criteria) and the supplementary file 2 (the 190 criteria and their 472 references). Discrepancies were found for 49 STOPP.v3 criteria, among which 17 with potential impact on the application of the criteria (e.g. A1: « without a clinical indication» instead of “without an evidence-based indication”; C5: removal of « without a clear indication for anticoagulation therapy» at the end of the criterion; C9: « longer than 12 months» instead of longer than 6 months). No discrepancy was observed between files 1 and 2 regarding the START criteria. The research team opted to further proceed with file 1.

Second, the two investigators carefully compared the SS.v3 criteria to the SS.v2 ones in order to identify the new ones and those with clinically significant modifications (i.e. criteria may change the prescribing practice). They independently categorized the 190 SS.v3 criteria from file 1 in three categories, i.e. the new, the modified and the unchanged criteria. Subdivision of criteria, or addition of examples were considered as unchanged criteria.

### Design of a user-friendly version of the criteria as a single-entry table

For user-friendliness, the scattering of a medication in various sections of the STOPP list [1] was solved by classifying the medication of each of the 133 STOPP criteria in its pharmacological class which was placed in its pharmacological section. A medication single-entry table was created, showing the code of each criterion to allow the clinicians to easily identify it and read its full text in the original article [1]. The single entry table gathers all the criteria applicable to each medication into a single row. This means each medication is represented once in the table, and all relevant criteria related to this medication are grouped together. Similarly, to provide a single entry, the medical condition or a concomitant treatment triggering each START criterion was classified under its main system. The START criteria were reordered to begin with the medical condition and then proceed to the medication, reflecting clinical practice where clinicians start from the patient's medical condition to identify potentially prescribing omissions (PPOs). The thirteen STOPP sections (A to M) and twelve START sections (A to L) were ordered by decreasing prevalence of PIMs and PPOs in geriatric patients [14–16, 23, 25, 26].

## Results

### STOPP.v3 criteria

#### New, modified and unchanged STOPP criteria

**Fig. 1:**
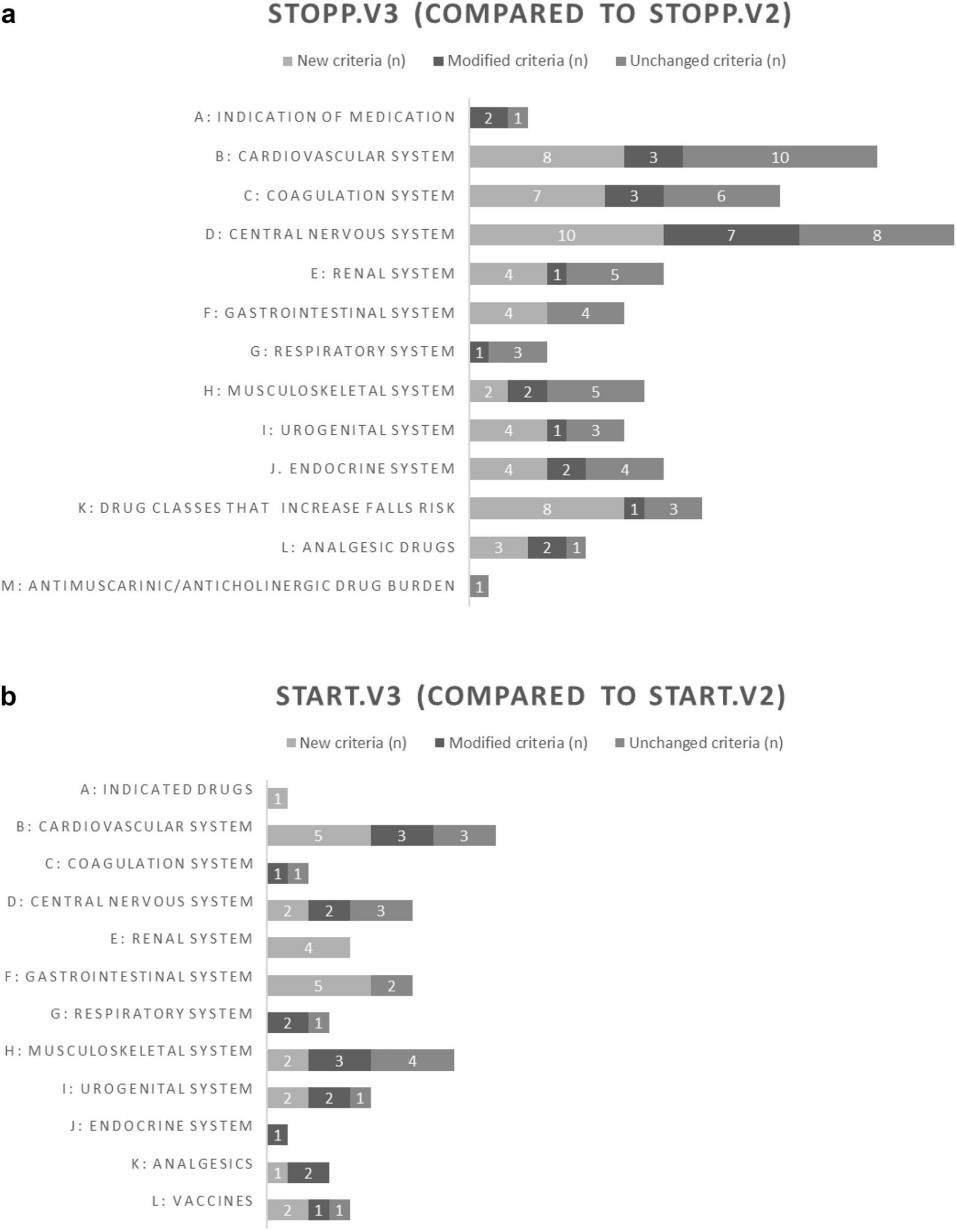
**a** New (n = 54), modified (n = 54) and unchanged (n = 54) STOPP criteria in SSv3 compared to SSv2. **b** New (n = 24) modified (n = 17) and unchanged START (n = 16) criteria in SSV3 compared to SSv2

The 133 STOPP.v3 criteria, compared to the 81 STOPP.v2 ones, were categorized into new (n = 54, 41%), modified (n = 25, 19%), and unchanged (n = 54, 41%) ones (Fig. 1a and Appendix 1a).

The 54 new STOPP criteria concern ten of the thirteen sections (Fig. 1a), with a majority (33/54) classified in four sections, namely the cardiovascular, coagulation, central nervous systems and fall-risk inducing drugs. New cardiovascular criteria (section B) include medications frequently prescribed in older adults, such as a beta-blocker as monotherapy for uncomplicated hypertension (B5), medications prolonging the QTc interval in patients with QTc prolongation (B15), a statin for primary cardiovascular prevention in patients ≥ 85 years with frailty and < 3 years of life expectancy (B16), and digoxin as first line treatment for long term (≥ 3 months) ventricular rate control in atrial fibrillation (B21). Of note, B14 and B17-19 (phosphodiesterase type-5 inhibitors, NSAIDS, antipsychotics, and corticosteroids) are not cardiovascular medications. Among the new coagulation STOPP criteria are vitamin K antagonist as first line anticoagulant for atrial fibrillation (C11) and P-glycoprotein (P-gp) drug efflux pump inhibitors making a direct oral anticoagulant (DOAC) potentially inappropriate (C14). Selective Serotonin Reuptake Inhibitor (SSRIs; C12) are included in the coagulation section due to their antiplatelet effect, reminded in D7. Oestrogens (C15) belong to the new criteria but are not coagulation medications. The new central nervous system criteria include a serotonin and norepinephrine reuptake inhibitors (SNRI) when severe hypertension (D3), a SSRI when significant bleeding (D7), a benzodiazepine (D10) and a Z-drug (D11) during ≥ 2 weeks for sleep problems. Six new criteria belong to the fall risk inducing drugs (FRIDs), involving anti-epileptic, first generation anti-histamine, opioid, anti-depressant, alpha-blocker, centrally-acting antihypertensive and anticholinergic drugs (K5-12). The renal section presents four new drugs/classes which belong to the endocrine (E7), urogenital (E8) and musculoskeletal (E9-10) systems. The endocrine section now points to sodium/glucose cotransporter 2 (SGLT2) inhibitors when symptomatic hypotension coexists (J4) and to levothyroxine for subclinical hypothyroidism (J9). Potentially inappropriate analgesic medications now include lidocaine patch for chronic osteoarthritis pain (L4) and gabapentinoids for non-neuropathic pain (L5).

The 25 STOPP.v3 criteria with modifications are identified in Appendix 2a (∆ symbols). Importantly, the first criterion (A1) is modified with « clinical indication» replacing « evidence-based indication». On the cardiovascular side, some situations are no longer potentially inappropriate in heart failure, e.g. verapamil/diltiazem in New York Heart Association NYHA III-IV Heart failure with preserved ejection fraction (HFpEF) (B2), a loop diuretic for arterial hypertension with concurrent heart failure (HF, B7) and NSAIDS unless the patient requires loop diuretic therapy (B19). Digoxin is now potentially inappropriate (E1) when at 125 µg/day or more when long-term (> 90 days) in patients with severe renal failure, i.e. estimated glomerular filtration rate (eGFR) < 30 ml/min/1.73 m^2^). As far as coagulation is concerned, two significant modifications are related to antiplatelet agents. The maximal dosage of long-term aspirin is reduced from 160 to 100 mg/day (C1). An antiplatelet agent is no longer potentially inappropriate in combination with an anticoagulant agent (C4) in patients with coronary artery disease (stent or > 50% stenosis) and atrial fibrillation. Regarding the central nervous system section, STOPP.v3 presents several modifications about tricyclic antidepressants (D1-2), antipsychotics (D4-5,15,21), and medications having potent anticholinergic effects (D14). In the urogenital section, silodosin is noted as not being potentially inappropriate when orthostatic hypotension is present. (I5). The modification in the analgesic section extends to moderate the level of break-through pain where long-acting opioid is prescribed in the absence of short-acting opioid (L3). This latter criterion might also belong to the START list. We similarly recommend converting two additional STOPP criteria into START criteria: the STOPP criterion F5 which points corticosteroids with history of peptic ulcer in the absence of proton pump inhibitor (PPI) and the STOPP criterion L2 which refers to opioids without laxatives. The STOPP criterion in version 2 on NSAID with PPI (C2) has logically become a START one in version 3 (F3).

### Single-entry STOPP table

The medication single-entry table (Table 1) presents 11 STOPP sections, with central nervous and coagulation PIMs listed first as they are the most prevalent ones in older people. Medications mentioned in several sections of SS.v3 are presented together. Table 1 presents several cases of single-entry medications found in various sections, e.g. benzodiazepine (D8-10; G4; K1), Z-drug (D11; K4), oral anticoagulants (C2; F6) and DOACs (C13-14; E2-3), SSRIs (D6-7; C12; K8), beta-blockers (B3-5; J3), digoxin (B1,4,21; E1) and NSAIDs (B17,19; C10; E4; H1-3,6,7). The medications of the renal section are distributed in the section concerned by their pharmacological action (e.g. digoxin in the cardiovascular system). The STOPP tool presents the 133 STOPP.v3 criteria in 58 single-entry medications in two pages (Table 1).

**Table 1:**
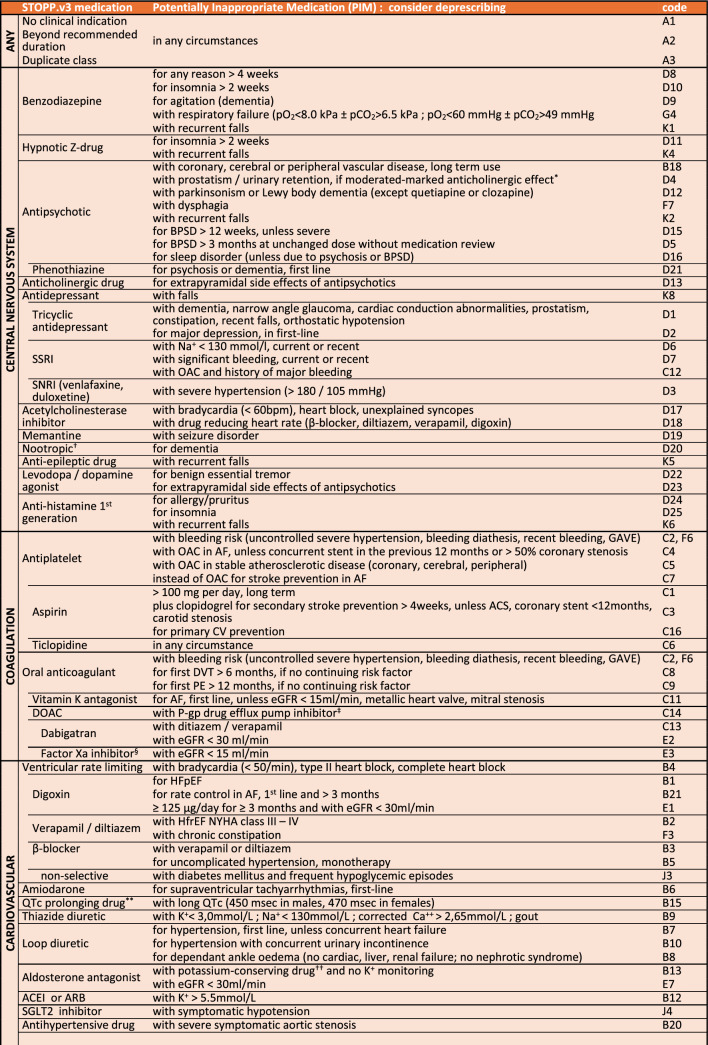

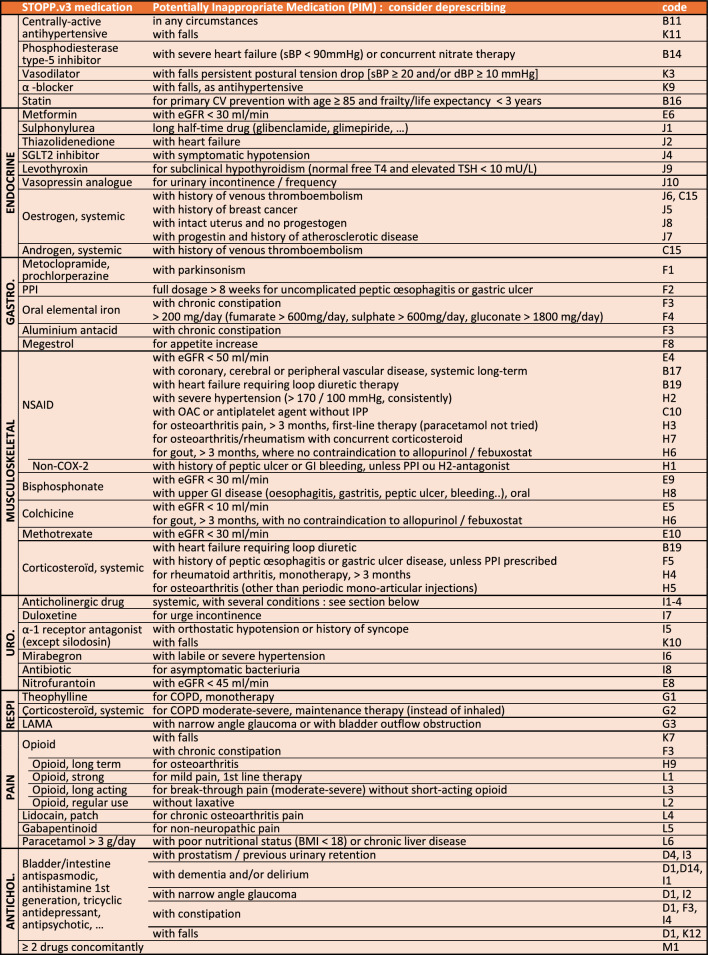
Single-entry STOPP.v3 criteria

Abbreviations: *ACEI* Angiotensin-converting-enzyme inhibitors, *ACS* Acute coronary syndrome, *AF* Atrial fibrillation, *ARB* Angiotensin II receptor blocker, *BMI* Body mass index, *BP* Blood pressure, *BPSD* Behavioral and psychological symptoms in dementia, *CKD* Chronic kidney disease,, *COPD* Chronic obstructive pulmonary disease, *COX-2* Cyclooxygenase-2, *CV* Cardiovascular, *dBP* diastolic blood pressure, *DMARD* Disease modifying activity rheumatic drug, *IV* Intravenous, *DOAC* Direct oral anticoagulant, *DVT* Deep venous thrombosis, *eGFR* estimated glomerular filtration rate, *GAVE* Gastric antral vascular ectasia, *GI* Gastro-intestinal, *HFpEF* Heart failure with preserved ejection fraction, *HFrEF* Heart failure with reduced ejection fraction, *LABA* Long-acting beta-agonists, *LAMA* Long-acting muscarinic antagonists, *NSAID* Nonsteroidal anti-inflammatory drugs, *NYHA* New York Heart Association, *OAC* Oral anticoagulant, *pCO2* partial pressure of carbon dioxide, *pO2* partial pressure of oxygen, *pa02* partial pressure of arterial oxygen, *PE* Pulmonary embolism, *PIM* Potentially inappropriate medication, *P-gp* P-glycoprotein, *PPI* Proton pump inhibitor, *PTH* Parathormone, *QTc* Corrected QT Interval, SaO2 arterial oxygen saturation, *SARS-CoV2* Severe acute respiratory syndrome coronavirus 2, *sBP* systolic blood pressure, *SGLT2* Sodium-glucose cotransporter 2, *SNRI* Serotonin–norepinephrine reuptake inhibitors, *SSRI* Selective Serotonin Reuptake Inhibitor, *T4* Thyroxine, *TSH* Thyroid stimulating hormone, *VKA* Vitamin K antagonist

^a^acepromazine, chlorpromazine, clozapine, flupenthixol, fluphenzine, levomepromazine, olanzapine, pipothiazine, promazine, thioridazine

^b^including Ginkgo Biloba, piracetam, pramiracetam, phenylpiracetam, aniracetam, phosphatidylserine, modafinil, L-theanine, omega-3 fatty acids, panax ginseng, rhodiola, creatine

^c^amiodarone, azithromycin, carvedilol, cyclosporin, dronedarone, itraconazole, ketoconazole (systemic), macrolides, quinine, ranolazine, tamoxifen, ticagrelor, verapamil

^d^rivaroxaban, apixaban, edoxaban

^e^quinolones, macrolides, ondansetron, citalopram (doses > 20 mg/day), escitalopram (doses > 10 mg/day), tricyclic antidepressants, lithium, haloperidol, digoxin, class 1A antiarrhythmics, class III antiarrhythmics, tizanidine, phenothiazines, astemizole, mirabegron

^f^ACEI’s, ARB’s, amiloride, triamterene

### START.v3 criteria

#### New, modified and unchanged START criteria

Three new START sections are present, namely sections A (indicated medications), C (coagulation) and E (renal). The STOPP and START system sections (B-J) are now presented in parallel. The 57 START.v3 criteria, when compared to the 34 START.v2 ones, were categorized in three groups, namely the new (n = 24, 42%), modified (n = 17, 30%) and unchanged (n = 16, 28%) criteria (Fig. 1b and Appendix 1b, Fig. 1b). All sections except for the coagulation (which is new but does not contain new criteria), the endocrine and the respiratory ones are concerned by new START criteria.

Most of the new START criteria (14/24) belong to the cardiovascular (B), renal (E) and gastro-intestinal (F) systems. Among the new cardiovascular criteria are three evidence-based medications for heart failure, i.e. spironolactone when eGFR > 30 ml/min (B7), SGLT2 when symptomatic (B8), and sacubitril/valsartan when there is persisting failure in the presence of reduced ejection fraction (B9, with angiotensin-converting-enzyme inhibitors (ACEI) withdrawal). The new coagulation section lists the two main medication classes (which belonged in SS.v2 to the cardiovascular section), i.e. the anticoagulant therapy for atrial fibrillation (C1) and the antiplatelet therapy for atherosclerotic disease (C2). The new renal section lists three cases of PPOs in severe renal failure, i.e. calcitriol, phosphate binder and erythropoietin analogue (E1-3).

The Appendix 2b lists the 17 START.v3 criteria with modifications (∆ symbols). Moderate to severe frailty has now to be taken into account in two START criteria i.e. antihypertensive therapy (initiation or intensification) when systolic blood pressure is above 140 mmHg (B1) and statin therapy for secondary cardiovascular prevention (B2). Regarding the respiratory system, the long-acting bronchodilators, muscarinic antagonists and beta-2 agonists, are now present (G1). The severity of chronic obstructive pulmonary disease and of chronic asthma is introduced (G1 *vs* G2). In the musculoskeletal system, calcium is no longer present in the drug therapy of osteoporosis (H3), except with long-term systemic corticosteroid therapy (H2). A confirmation of vitamin D deficiency is now required in the vitamin D criterion for patients who are housebound, experiencing falls and/or presenting osteopenia (H5). The endocrine section has a single and modified criterion, an ACEI (or Angiotensin II Receptor Blockers if intolerance) when proteinuria is present in a patient with diabetes (J1). This medication however belongs to the cardiovascular system.

### Single-entry START table

The ten START sections present musculoskeletal and cardiovascular placed first (Table 2). The START tool presents the 57 START.v3 criteria in 48 single-entry medical conditions in one page.

**Table 2:**
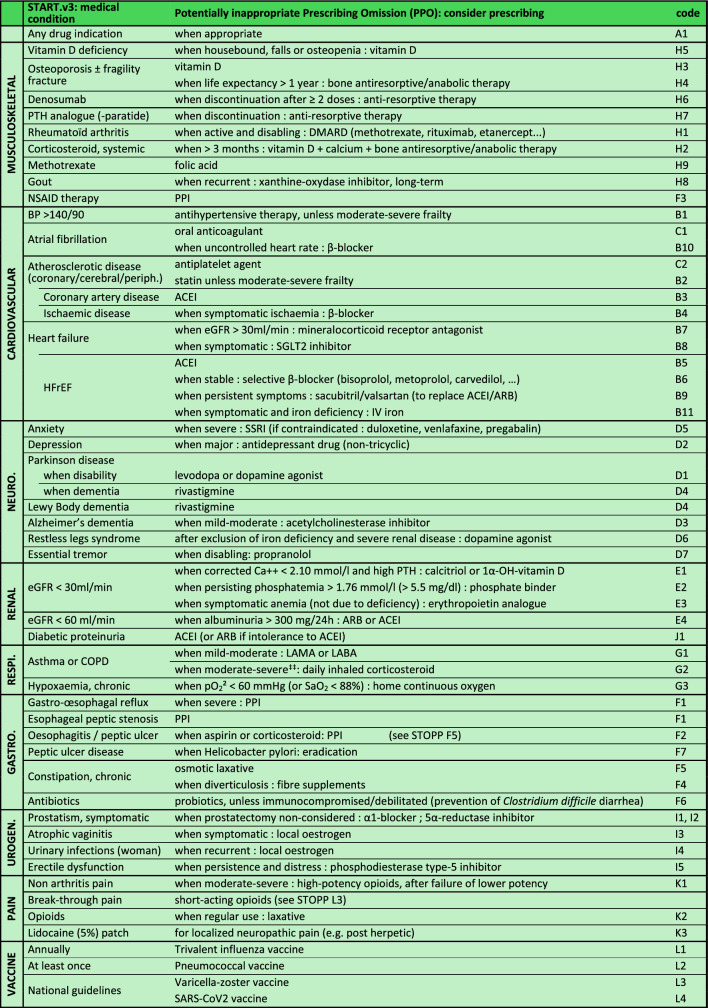
Single-entry START.v3 criteria

Abbreviations: *ACEI* Angiotensin-converting-enzyme inhibitors, *ACS* Acute coronary syndrome, *AF* Atrial fibrillation, *ARB* Angiotensin II receptor blocker, *BMI* Body mass index, *BP* Blood pressure, *BPSD* Behavioral and psychological symptoms in dementia, *CKD* Chronic kidney disease,, *COPD* Chronic obstructive pulmonary disease, *COX-2* Cyclooxygenase-2, *CV* Cardiovascular, *dBP* diastolic blood pressure, *DMARD* Disease modifying activity rheumatic drug, *IV* Intravenous, *DOAC* Direct oral anticoagulant, *DVT* Deep venous thrombosis, *eGFR* estimated glomerular filtration rate, *GAVE* Gastric antral vascular ectasia, *GI* Gastro-intestinal, *HFpEF* Heart failure with preserved ejection fraction, *HFrEF* Heart failure with reduced ejection fraction, *LABA* Long-acting beta-agonists, *LAMA* Long-acting muscarinic antagonists, *NSAID* Nonsteroidal anti-inflammatory drugs, *NYHA* New York Heart Association, *OAC* Oral anticoagulant, *pCO2* partial pressure of carbon dioxide, *pO2* partial pressure of oxygen, *pa02* partial pressure of arterial oxygen, *PE* Pulmonary embolism, *PIM* Potentially inappropriate medication, *P-gp* P-glycoprotein, *PPI* Proton pump inhibitor, *PTH* Parathormone, *QTc* Corrected QT Interval, SaO2 arterial oxygen saturation, *SARS-CoV2* Severe acute respiratory syndrome coronavirus 2, *sBP* systolic blood pressure, *SGLT2* Sodium-glucose cotransporter 2, *SNRI* Serotonin–norepinephrine reuptake inhibitors, *SSRI* Selective Serotonin Reuptake Inhibitor, *T4* Thyroxine, *TSH* Thyroid stimulating hormone, *VKA* Vitamin K antagonist

^a^where FEV1 <50% of predicted value and repeated exacerbations requiring treatment with oral corticosteroids

## Discussion

This contribution to the STOPP/START version.3 identifies 78 new criteria as well as 42 criteria with significant modifications (Appendices 1 and 2). It provides the clinicians with a user-friendly tool designed to facilitate medication review in daily practice (Tables 1 and 2).

In the updated SS.v3 [1], the structure of the STOPP sections remains unchanged, while START sections have been expanded. The STOPP and START system sections (B-J) are now presented in parallel. The STOPP/START.v3 lists include numerous new criteria, mainly in medications classes frequently encountered in older patients. The addition of some criteria aligns with recent guidelines updates and significant advancements in clinical practice (e.g. SGLT2 inhibitors, the distinction between heart failure with preserved or reduced ejection fraction, vaccination) which is a welcome development. Another notable feature of the version 3 criteria is the inclusion of geriatric syndromes both in STOPP (e.g. frailty, behavioural and psychological symptoms of dementia, delirium, dysphagia) and in START (frailty, functional impairment, chronic constipation) [5]. The clinically important fall-risk inducing drugs (FRIDs) criteria now include eight new medications.

The present work may be useful for experts to evaluate how the new and modified criteria align with recommendations in their country, region, or practice, and to discuss their validity and applicability. The clear identification of new and modified criteria is not only valuable for users of SS.v2 but also for those developing electronic clinical decision system (CDS) tool based on the STOPP/START criteria. The conversion of the list of criteria into a single-entry table is an intermediate step in preparing a CDS. The creation of rules requires identifying components relevant to operationalization [27], and the single-entry table aids in this process. Finally, the CDS results should be presented to users concisely. We believe that grouping the various reasons why a medication is inappropriate on a single screen would be an effective way to alert the prescriber.

We designed a user-friendly version of the criteria, featuring a single entry for each medication (STOPP) and clinical condition (START). Each START criterion is initiated with its corresponding medical condition, not the medication. This approach aligns with the clinical process, which examines medical conditions to identify instances where a medication may have been omitted.

This work aligns with recent efforts to enhance the usability of the Beers criteria [13]. The Beers criteria are also presented in a table format, with the order of the criteria related to the systems. The panel responsible for developing the latest Beers criteria focused on the order and wording of criteria to improve usability [13]. However, the Beers criteria consist of several lists, with the one linking medications and conditions presented with the condition first, as a single entry. In contrast, our presentation of the STOPP criteria prioritizes the drug first, as this approach was preferred during continuing education workshops we conducted on medication review.

Using a similar format facilitates the comparison of criteria from different sources. We believe that presenting criteria as a single-entry table should be promoted. We expect this paper to pave the way for greater uniformity in clinical decision support tools.

This tool represents an update and translation from French to English of our work on the SS.v2, published in 2015 [24]. Our clinical and teaching experiences since 2015 indicate that the single-entry format streamlines the medical review process. This is particularly true for frequently prescribed medications that belong to multiple sections. Initially, based on our research findings on SS.v1, we designed a list that collected, on a single line, all the PIMs associated with each of the 10 most frequent STOPP drugs and all the PPOs associated with each of the 10 most frequent START medical conditions. As mentioned above, these "single-entry" short lists were used in 2011 as the cornerstone of several workshops on medication review, gathering hundreds of Belgian general practitioners (GPs) in continuing medical education. The survey completed by these GPs after the training showed a high level of satisfaction with the "single-entry" approach.

In 2015, following the publication of SS.v2, we extended the single-entry tables to include all 115 criteria. We used the tool during continued education workshops on medication review, gathering more than 150 GPs in Belgium and Luxembourg, with positive feedback on this approach. Additionally, we tested its usability with 60 young Belgian physicians in GP training. Their satisfaction was very high (72%) and high (26%) regarding its didactic value, and very high (84%) and high (16%) regarding its potential impact on daily practice (unpublished data).

We then published the single-entry table in French in Louvain Médical, the medical journal of UCLouvain [24]. Since then, it has been further disseminated nationnaly during symposiums, conferences, and meetings. The SS.v2 single-entry table is now part of the UCLouvain program for medical and pharmacy students. It is also part of our clinical practice as pharmacists and geriatricians, and we train our interns in its use.

The format is user-friendly for all users, and especially beneficial for new users of these criteria. The process of adopting new recommendations is extensive, and the emphasis on user-friendliness is crucial. We anticipate that researchers, clinicians, and students will embrace our formatted tool, thereby enhancing the positive impact of SS on the care of older patients. Conducting usability testing of this format could further promote its adoption. One could compare the performance of medication reviews using the original list versus the single-entry table tool in terms of inappropriate prescription (IP) detection, time efficiency, and user satisfaction. This could be carried out in primary and secondary care, by physicians, pharmacists and other healthcare professionals engaged in medication reviews.

In conclusion, this work might assist clinicians, students and researchers in more seamlessly adopting the new SS.v3 criteria. By presenting these criteria in an easily accessible single-entry table, we hope to facilitate their integration into daily clinical practice.

## Supplementary Information

Below is the link to the electronic supplementary material.

Supplementary file1 (DOCX 49 KB)
