# Supplementary material for: Improving usability of the STOPP/START version 3 criteria: development of a practice tool for clinicians, students and researchers
Source: Eur Geriatr Med. 2025 Jun 17;16(4):1403–13. doi: 10.1007/s41999-025-01214-y (PMC12378565; doi:10.1007/s41999-025-01214-y)
Supplement: Supplementary file 1 — Supplementary file1 (DOCX 49 KB) [file 41999_2025_1214_MOESM1_ESM.docx]

# Appendices to

# Improving usability of the STOPP/START version 3 criteria: development of a practice tool for clinicians, students and researchers.

O Dalleur ^1-2-3^, FX Sibille ^1-2-4^, A Mouzon ^5^,

F Vaillant ^3^, S Marien ^1-2-4^, A Spinewine ^1-2-5^, B Boland ^2-6^

Affiliations

^1^ Clinical Pharmacy & Pharmacoepidemiology research group, Louvain Drug Research Institute, UCLouvain, Brussels, Belgium

^2^ Groupe de Recherche en Gériatrie & Gérontologie, Institut de Recherche Santé et Société (IRSS), UCLouvain, Belgium

^3^ Pharmacy department, Cliniques universitaires Saint-Luc, Brussels, Belgium

^4^ Geriatric Medicine department, CHU UCL Namur, Yvoir, Belgium

^5^ Pharmacy department, CHU UCL Namur, Yvoir, Belgium

^6^ Geriatric Medicine, Cliniques universitaires Saint-Luc, Brussels, Belgium

Address for correspondence

[olivia.dalleur@uclouvain.be](mailto:olivia.dalleur@uclouvain.be)

Appendix 1a Comparison of STOPP.v2 and STOPP.v3 by section

| Section | Criteria | New criteria | Modified criteria * | Unchanged criteria |
| --- | --- | --- | --- | --- |
| Total | n = 133 | n = 54 (41%) | n = 25 (19 %) | n = 54(41 %) |
| A. Indication | n = 3 |  | A1,3 | A2 |
| B. Cardiovascular | n = 21 | B5,15,16,17,18,19,20,21 | B2,4,7 | B1,3,6,8,9,10,11,12,13,14 |
| C. Coagulation | n = 16 | C7,11,12,13,14,15,16 | C1,3,4 | C2,5,6,8,9,10 |
| D. Central nervous | n = 25 | D3,5,7,9,10,11,19,20,23,25 | D1,2,14,15,18,21,24 | D4,6,8,12,13,16,17 ,22 |
| E. Renal | n = 10 | E7,8,9,10 | E1 | E2,3,4,5,6 |
| F. Gastrointestinal | n = 8 | F5,6,7,8 |  | F1,2,3,4 |
| G. Respiratory | n = 4 |  | G3 | G1,2,4 |
| H. Musculo-skeletal | n = 9 | H7,9 | H2,8 | H1,3,4,5,6 |
| I. Urogenital | n = 8 | I4,6,7,8 | I5 | I1,2,3 |
| J. Endocrine | n= 10 | J4,7,9,10 | J3,8 | J1,2,5,6 |
| K. Fall-risk drugs | n= 12 | K5,6,7,8,9,10,11,12 | K3 | K1,2,4 |
| L. Analgesic drugs | n= 6 | L4,5,6 | L2,3 | L1 |
| M. Anticholinergic | n= 1 |  |  | M1 |

* clinically significant modifications (changes in prescribing practice)

Appendix 1b. Comparison of START.v2 and START.v3 by section

| Section | Criteria | New criteria | Modified criteria * | Unchanged criteria |
| --- | --- | --- | --- | --- |
| Total | n = 57 | n = 24 (42%) | n = 17 (30%) | n = 16 (28%) |
| A. Indication | n = 1 | A1 |  |  |
| B. Cardiovascular | n = 11 | B7,8,9,10,11 | B1,2,5 | B3,4,6 |
| C. Coagulation | n = 2 |  | C1 | C2 |
| D. Central nervous | n = 7 | D4,7 | D2,5 | D1,3,6 |
| E. Renal | n = 4 | E1,2,3,4 |  |  |
| F. Gastrointestinal | n = 7 | F2,3,5,6,7 |  | F1,4 |
| G. Respiratory | n = 3 |  | G1,2 | G3 |
| H. Musculoskeletal | n = 9 | H6,7 | H3,4,5 | H1,2,8,9 |
| I. Urogenital | n = 5 | I4,5 | I1,2 | I3 |
| J. Endocrine | n = 1 |  | J1 |  |
| K. Analgesics | n = 3 | K3 | K1,2 |  |
| L. Vaccines | n = 4 | L3,4 | L1 | L2 |

* clinically significant modifications (changes in prescribing practice)

Appendix 2a. STOPP.v3 criteria.

**Medication in new** criteria in bold. Criteria with significant *modifications* are pointed by *∆* (modifications in italic). ~~Removed~~ in SS.v3

{risk associated with the PIM}

Section A: Drug indication

*∆* A1*.* Any drug prescribed without ~~evidence-based~~ *clinical indication.*

A2. Any drug prescribed beyond the recommended duration, where treatment duration is well defined.

*∆* A3*.* Any duplicate drug class prescription *for daily regular use* *(as distinct from PRN use)* [e.g. two concurrent NSAIDs, SSRIs, loop diuretics, ACEI, anticoagulants, *antipsychotics, opioid analgesics]* {optimisation of monotherapy within a single drug class should be observed prior to considering a new agent}

Section B: Cardiovascular System

B1. Digoxin for heart failure with preserved ejection fraction (HFpEF) (no clear evidence of benefit)

*∆* B2*.* Verapamil/diltiazem with NYHA Class III or IV heart failure *with reduced ejection fraction* *(HFrEF)* {worsening}

B3. Beta-blocker with verapamil or diltiazem {heart block}

*∆* B4*. Ventricular rate-limiting drugs* [i.e. beta-blocker, verapamil, diltiazem, digoxin] with bradycardia (< 50/min), type II heart block or complete heart block {profound hypotension, asystole}

**B5. Beta-blocker** as monotherapy for uncomplicated hypertension [i.e. not associated with angina pectoris, aortic aneurysm or other condition where beta-blocker therapy is indicated] (no firm evidence of efficacy)

B6. Amiodarone as first-line antiarrhythmic therapy in supraventricular tachyarrhythmias {major side effects than beta-blockers, digoxin, verapamil or diltiazem}

*∆* B7*.* Loop diuretic as first-line treatment for hypertension *unless there is concurrent heart failure requiring diuretic therapy* (*lack of outcome data for this indication*; safer, more effective alternatives available)

B8. Loop diuretic for dependent ankle oedema without clinical, biochemical evidence or radiological evidence of heart failure, liver failure, nephrotic syndrome or renal failure (leg elevation and/or compression hosiery more appropriate)

B9. Thiazide diuretic with current significant hypokalaemia [i.e. serum K+ < 3.0 mmol/l], hyponatraemia [i.e. serum Na+ < 130 mmol/l], hypercalcaemia [i.e. corrected serum calcium > 2.65 mmol/l] or with a history of gout {hypokalaemia, hyponatraemia, hypercalcaemia and gout can be precipitated by thiazide diuretic}

B10. Loop diuretic for treatment of hypertension with concurrent urinary incontinence {may exacerbate incontinence}

B11. Centrally-acting antihypertensive [e.g. methyldopa, clonidine, moxonidine, rilmenidine, guanfacine] (centrally-active antihypertensives are generally less well tolerated by older people than younger people)

B12. ACEIACEI or ARB with hyperkalaemia *[i.e. K+ > 5.5].*

B13. Aldosterone antagonist [e.g. spironolactone, eplerenone] with concurrent potassium-conserving drugs [e.g. ACEI, ARB, amiloride, triamterene] without monitoring of serum K+ {dangerous hyperkalaemia [K+ > 6.0} (K+ monitoring < 6 months)

B14. Phosphodiesterase type-5 inhibitor [e.g. sildenafil, tadalafil, vardenafil] with severe heart failure (systolic BP < 90 mmHg] or with concurrent nitrate therapy for angina {cardiovascular collapse}

**B15. Drugs prolonging the QTc interval** (QTc = QT/RR with known QTc prolongation (> 450 msec in males, > 470 msec in females) [e.g. quinolone, macrolide, ondansetron, citalopram (>20 mg/d), escitalopram (>10 mg/d), tricyclic antidepressant, lithium, haloperidol, digoxin, class IA antiarrhythmic, class III antiarrhythmic, tizanidine, phenothiazine, astemizole, mirabegron] {life-threatening ventricular arrhythmias}

**B16. Statin** for primary cardiovascular prevention with age ≥ 85 years and established frailty / expected life expectancy < 3 years (lack of evidence of efficacy)

**B17. NSAID long-term,** systemic, with known history of coronary, cerebral or peripheral vascular disease {thrombosis}

**B18. Antipsychotic** long term with known history of coronary, cerebral or peripheral vascular disease {thrombosis}

**B19. NSAID or corticosteroid**, systemic, with heart failure requiring loop diuretic therapy {risk of HFailure exacerbation}

**B20. Antihypertensive drug** (except ACEI and ARB) with severe symptomatic aortic stenosis {severe hypotension, syncope}

**B21. Digoxin** as first line treatment for long-term [> 3 months] ventricular rate control in atrial fibrillation {increased mortality from long-term digoxin use} (cardio-selective beta-blockers are generally preferable)

Section C: Coagulation System

*∆* C1. Long-term aspirin at doses > *100 mg* per day (no evidence for increased efficacy) {bleeding}

C2. Antiplatelet agents or anticoagulant [i.e. vitamin K antagonist, direct thrombin inhibitor, factor Xa inhibitor] with concurrent significant bleeding risk [i.e. uncontrolled severe hypertension, bleeding diathesis, recent non-trivial spontaneous bleeding] {bleeding}

*∆* C3*.* Aspirin plus clopidogrel as *long-term [i.e. > 4 weeks]* secondary stroke prevention, unless the patient has a coronary stent(s) inserted in the previous 12 months or concurrent acute coronary syndrome or has a high grade symptomatic carotid arterial stenosis (no evidence of long-term benefit over clopidogrel monotherapy) {bleeding}

*∆* C4*.* Antiplatelet and anticoagulant [i.e. vitamin K antagonist, direct thrombin inhibitor, factor Xa inhibitor] with chronic atrial fibrillation *unless there is concurrent coronary artery stent(s) inserted or angiographically proven high grade (> 50%) coronary artery stenosis* (no added benefit from antiplatelet agents) {bleeding}

C5. Antiplatelet and anticoagulant [i.e. vitamin K antagonist, direct thrombin inhibitor or factor Xa inhibitor] with stable coronary, cerebrovascular or peripheral arterial disease (no added benefit from dual therapy) {bleeding}

C6. Ticlopidine in any circumstance (clopidogrel and prasugrel have similar efficacy, stronger evidence and fewer side effects)

**C7. Antiplatelet** as alternative to anticoagulant [i.e. vitamin K antagonist, direct thrombin inhibitor, factor Xa inhibitor] for stroke prevention in patients with chronic atrial fibrillation (no evidence of efficacy)

C8. Anticoagulant [vitamin K antagonist, direct thrombin inhibitor, factor Xa inhibitor] > 6 months for first deep venous thrombosis without continuing provoking risk factor (no proven added benefit)

C9. Anticoagulant [i.e. vitamin K antagonist, direct thrombin inhibitor, factor Xa inhibitor) > 12 months for first pulmonary embolus without continuing provoking risk factor (no proven added benefit)

C10. NSAID with anticoagulant [i.e. vitamin K antagonist, direct thrombin inh., factor Xa inhibitor] {*major* gastrointestinal bleeding}

**C11. Vitamin K antagonist** as first-line anticoagulant for atrial fibrillation, unless there is concurrent metallic heart valve in-situ, moderate-severe mitral stenosis, or creatinine clearance < 15ml/min (DOAC equally efficacious and safer than vitamin K antagonist)

**C12. SSRI** (selective serotonin reuptake inhibitor) with anticoagulant (vitamin K antagonist, direct thrombin inhibitor, factor Xa inhibitor) with history of major haemorrhage {bleeding due to antiplatelet effects of SSRI}

**C13. Dabigatran** (direct thrombin inhibitor) with diltiazem or verapamil {bleeding}

**C14. DOAC** [i.e. apixaban, dabigatran, edoxaban, rivaroxaban] with P-glycoprotein (P-gp) drug efflux pump inhibitor [e.g. amiodarone, azithromycin, carvedilol, cyclosporin, dronedarone, itraconazole, ketoconazole (systemic), macrolides, quinine, ranolazine, tamoxifen, ticagrelor, verapamil] {bleeding}

**C15.** Oestrogen or **androgen**, systemic, with history of venous thromboembolism {recurrent venous thromboembolism}

**C16. Aspirin** for primary prevention of cardiovascular disease.

Section D: Central Nervous System

*∆* D1*.* Tricyclic antidepressant with dementia, narrow angle glaucoma, cardiac conduction abnormalities, lower urinary tract symptoms related to benign prostatic hyperplasia or prior history of urinary retention, *chronic constipation, recent falls* or *orthostatic hypotension* {worsening}

*∆* D2*.* Initiation of tricyclic antidepressant as first-line treatment for *major depression* {more adverse drug reactions than with SSRI or SNRI}

**D3. SNRI** (serotonin/noradrenaline reuptake inhibitors) [e.g. venlafaxine, duloxetine] with severe hypertension [i.e. systolic blood pressure > 180 mmHg ± diastolic blood pressure > 105 mmHg] {hypertension worsening}

D4. Antipsychotic having *moderate*-marked anticholinergic effects [i.e. *acepromazine*, chlorpromazine, clozapine, flupenthixol, fluphenzine, levomepromazine, *olanzapine, pipothiazine, promazine, thioridazine*] with history of lower urinary tract symptoms associated with benign prostatic hyperplasia or previous urinary retention {urinary retention}

D5. **Antipsychotic** for behavioural and psychological symptoms of dementia *at unchanged dose for > 3 months without medication review* {extrapyramidal side effects chronic worsening of cognition, major cardiovascular morbidity and mortality}

D6. Selective serotonin re-uptake inhibitors (SSRI) with current or recent hyponatraemia [i.e. Na+ < 130] {hyponatraemia}

**D7. SSRI** with current or recent significant bleeding {bleeding due to antiplatelet effect}

D8. Benzodiazepine for ≥ 4 weeks, no indication for longer treatment {prolonged sedation, confusion, impaired balance, falls, road traffic accidents} (All benzodiazepines should be withdrawn gradually when taken for > 4 weeks as there is a risk of causing a benzodiazepine withdrawal syndrome if stopped abruptly)

**D9. Benzodiazepine** for agitated behaviour or non-cognitive symptoms of dementia (no evidence of efficacy)

**D10. Benzodiazepine** ≥ 2 weeks for insomnia {dependency, falls, fractures, traffic accidents}

**D11. Z-drug** [i.e. zolpidem, zopiclone, zaleplon] ≥ 2 weeks for insomnia {falls, fractures}

D12. Antipsychotic (except clozapine, quetiapine) with parkinsonism / Lewy body dementia {extra-pyramidal symptoms}

D13. Anticholinergic drug *[i.e. biperiden, ophenadrine, procyclidine, trihexyphenidyl]* for extra-pyramidal side effects of antipsychotic medications {anticholinergic toxicity}

*∆* D14. Drug having *potent* anticholinergic effects with delirium or dementia {exacerbation of cognitive impairment}

*Commonly prescribed drugs with potent anticholinergic effects include tricyclic antidepressants [e.g., amitriptyline, doxepin, impramine, nortriptyline], antipsychotics (chlorpromazine, clozapine, thioridazine), first generation antihistamines [e.g. diphenhydramine, chlorpheniramine], bladder anti-spasmodics [e.g., tolterodine, oxybutynin], hyoscine, procyclidine, benzatropine, tizanidine.*

*∆* D15. Antipsychotic *> 12 weeks* for non-cognitive symptoms of dementia unless symptoms are severe, while other treatments have failed {stroke, *myocardial infarction*}

D16. Antipsychotic as hypnotic, unless sleep disorder is due to psychosis or non-cognitive symptoms of dementia {confusion, hypotension, extra-pyramidal side effects, falls} (*not recommended in summary of product characteristics*)

D17. Acetylcholinesterase inhibitor with history of persistent bradycardia (< 60 beats/min.), heart block or recurrent unexplained

syncope {cardiac conduction failure, syncope and injury}

*∆* D18. Acetylcholinesterase inhibitor *with drugs* reducing heart rate [beta-blocker, digoxin, diltiazem, verapamil] {cardiac conduction failure, syncope and injury}

**D19. Memantine** with current or previous seizure disorder {seizures}

**D20. Nootropic** [e.g. Gingko Biloba, piracetam, pramiracetam, phenylpiracetam, aniracetam, phosphatidylserine, modafinil, L-theanine, omega-3 fatty acids, panax ginseng, rhodiola, creatine] for dementia (no evidence of efficacy)

*∆* D21. Phenothiazine first-line *for psychosis or non-cognitive symptoms of dementia* since safer and more efficacious alternatives exist {sedation, anti-cholinergic toxicity} (exceptions: chlorpromazine for persistent hiccoughs; prochlorperazine for nausea/vomiting/vertigo; levomepromazine for anti-emetic nausea/vomiting in palliative care}

D22. Levodopa or dopamine agonist for benign essential tremor (no evidence of efficacy)

**D23. Levodopa or dopamine agonist** for extrapyramidal side effects from antipsychotics or other forms of drug-induced Parkinsonism (inappropriate prescribing cascade)

*∆* D24. First-generation antihistamine agent first line *for allergy or pruritus* (safer, less toxic antihistamines widely available)

**D25. First-generation antihistamine agent** *for insomnia* *{high risk of side effects} (Z-drugs safer for short-term use)*

Section E. Renal System (eGFR in ml/min/1.73m²)

∆ E1. Digoxin long-term *(> 3 months)* *≥ 125* µg/day with eGFR < 30 {digoxin toxicity if plasma levels not measured}

E2. Direct thrombin inhibitor (dabigatran) with eGFR < 30 {bleeding}

E3. Factor Xa inhibitor [e.g. rivaroxaban, apixaban, *edoxaban*] with eGFR < 15 {bleeding}

E4. NSAID with eGFR < 50 {deterioration in renal function}

E5. Colchicine with eGFR < 10 {colchicine toxicity}

E6. Metformin with eGFR < 30 {lactic acidosis}

**E7. Mineralocorticoid receptor antagonist** [e.g. spironolactone, eplerenone] with eGFR<30 {dangerous hyperkalemia}

**E8. Nitrofurantoin** with eGFR < 45 {nitrofurantoin toxicity}

**E9. Bisphosphonate** with eGFR < 30 {acute renal failure}

**E10. Methotrexate** with eGFR < 30 {methotrexate toxicity}

Section F: Gastrointestinal System

F1. Prochlorperazine or metoclopramide with parkinsonism {exacerbation of Parkinsonian symptoms}

F2. PPI > 8 weeks at full dosage for uncomplicated peptic ulcer disease (dose reduction or earlier discontinuation *or H2 antagonist maintenance* usually indicated).

F3. Drug likely to cause constipation [e.g. antimuscarinic/anticholinergic drugs, oral iron, opioids, verapamil, aluminium antacids] with chronic constipation where non-constipating alternatives are appropriate {exacerbation of constipation}

F4. Oral elemental iron > 200 mg daily [e.g. ferrous fumarate > 600 mg/day, ferrous sulphate > 600 mg/day, ferrous gluconate > 1800 mg/day] (no evidence of enhanced iron absorption above these doses).

**F5. Corticosteroid** with a history of peptic ulcer disease or erosive oesophagitis {relapse, unless PPI is co-prescribed}

**F6. Antiplatelet or anticoagulant** with gastric antral vascular ectasia [“watermelon stomach”] {major gastrointestinal bleeding}

**F7. Antipsychotic** with dysphagia {aspiration pneumonia}

**F8. Megestrol acetate** for appetite {thrombosis and death with unproven efficacy}

Section G. Respiratory System

G1. Theophylline monotherapy for COPD (safer, more effective alternative) {adverse effects, due to narrow therapeutic index}

G2. Systemic corticosteroid instead of inhaled corticosteroids for maintenance therapy in moderate-severe COPD (effective inhaled therapies are available) {long-term side effects of systemic corticosteroids}

*∆* G3. *LAMA (long-acting* muscarinic antagonists) [e.g. tiotropium, *aclidinium*, *umeclidinium, glycopyrronium*] with narrow angle glaucoma {glaucoma exacerbation} or bladder outflow obstruction {urinary retention}

G4. Benzodiazepine with acute or chronic respiratory failure [i.e. pO² < 8.0 kPa ± pCO²>6.5 kPa] {respiratory failure exacerbation}

Section H: Musculoskeletal System.

H1. Non-COX-2 selective NSAID with history of peptic ulcer disease or gastrointestinal bleeding, unless with concurrent PPI or H2 antagonist {peptic ulcer relapse}

∆ H2. NSAID with severe hypertension [*i.e. sBP > 170 and/or dBP > 100 mmHg, consistently* {exacerbation of hypertension}

H3. NSAID long term (>3 months) for symptom relief of osteoarthritis pain where paracetamol has not been tried (simple analgesics preferable and usually as effective for pain relief)

H4. Corticosteroid long term (>3 months) as monotherapy for rheumatoid arthritis {systemic corticosteroid side effects}

H5. Corticosteroid for osteoarthritis (other than periodic mono-articular injections) {systemic corticosteroid side effects}

H6. NSAID (>3 months) or colchicine (>3 months) for *prevention of relapses of* gout where there is no contraindication to a xanthine-oxidase inhibitor [e.g. allopurinol, febuxostat] (xanthine-oxidase inhibitors are first prophylactic choice).

**H7. NSAID** with concurrent corticosteroids *for treatment of arthritis/rheumatism of any kind* {peptic ulcer disease}

∆ H8. Oral bisphosphonates with *current or recent* history of upper gastrointestinal disease [i.e. oesophagitis, gastritis, duodenitis, peptic ulcer disease, or upper gastrointestinal bleeding] {relapse/exacerbation}

**H9. Opioid** long-term for osteoarthritis (lack of evidence of efficacy) {serious side effects}

Section I: Urogenital System.

I1. Antimuscarinic drug*, systemic,* with dementia or chronic cognitive impairment {increased confusion, agitation}

I2. Antimuscarinic drug*, systemic,* with narrow-angle glaucoma {acute exacerbation of glaucoma}

I3. Antimuscarinic drug, *systemic,* with lower urinary tract symptoms associated with benign prostatic hyperplasia *and high post-void residual volume [i.e. > 200 ml]* (uncertain efficacy) {urinary retention in older men}

**I4. Antimuscarinic drug,** *systemic,* with constipation {exacerbation of constipation}

*∆* I5. Alpha-1 receptor antagonists *other than silodosin* [e.g. alfuzosin, doxazosin, indoramin, tamsulosin, terazosin] with symptomatic orthostatic hypotension or *history of syncope* {recurrent syncope}

**I6. Mirabegron** with labile or severe hypertension {exacerbation of hypertension}

**I7: Duloxetine** with urinary urgency or urge incontinence (drug indicated in stress incontinence but not in urge incontinence).

**I8. Antibiotic** use for asymptomatic bacteriuria (no indication for treatment).

Section J: Endocrine System.

J1. Sulphonylurea, long half-life [e.g. glibenclamide, chlorpropamide, glimepiride] for type 2 diabetes {prolonged hypoglycaemia}

J2. Thiazolidenedione [e.g. rosiglitazone, pioglitazone] with heart failure {exacerbation of heart failure}

∆ J3. *Non-selective* beta-blocker [i.e. other than bisoprolol, nebivolol, metoprolol, carvedilol] with diabetes mellitus and frequent hypoglycaemic episodes {suppression of hypoglycaemic symptoms}

**J4.** **SGLT2 inhibitor** (sodium glucose co-transporter inhibitor) [e.g. canagliflozin, dapagliflozin, empagliflozin, ertugliflozin] with symptomatic hypotension {exacerbation of hypotension}

J5. *Systemic* oestrogen with history of breast cancer {recurrence}

J6. *Systemic* oestrogen with history of venous thromboembolism {recurrence}

**J7. Menopausal hormone therapy** (oestrogen plus progestin) with stenotic coronary, cerebral or peripheral arterial disease {acute arterial thrombosis}

∆ J8. *Systemic* oestrogens without progestogens and intact uterus {endometrial cancer}

**J9. Levothyroxine** for subclinical hypothyroidism [i.e. normal free T4 and elevated TSH but < 10 mU/L] (no evidence of benefit) {iatrogenic thyrotoxicosis).

**J10. Vasopressin analogue** [e.g. desmopressin, vasopressin] for urinary incontinence or frequency {symptomatic hyponatraemia}.

Section K: Drugs that predictably increase the risk of falls in older people.

K1. Benzodiazepine *with falls* {daytime sedation, reduced sensorium, impair balance}

K2. Antipsychotic drug *with falls* {parkinsonism}

∆ K3. Vasodilator drug *with falls* or persistent postural tension drop [i.e. sBP ≥ 20 mmHg *and/or dBP ≥ 10 mmHg]* {syncope, falls}

K4*.* Hypnotic Z-drug [i.e., zopiclone, zolpidem, zaleplon] *with falls* {daytime sedation, ataxia}

**K5. Anti-epileptic drug** with falls {impairment of sensorium and cerebellar function}

**K6. First generation antihistamine** with falls {sensorium impairment}

**K7. Opioid** with falls {sensorium impairment}

**K8. Antidepressant** with falls {sensorium impairment}

**K9. Alpha-blocker as antihypertensives** with falls {orthostatic hypotension}

**K10. Alpha-blocker** for prostatic bladder outflow symptoms (other than silodosin) with falls {orthostatic hypotension}

**K11. Centrally acting antihypertensive** {sensorium impairment, orthostatic hypotension}

**K12. Antimuscarinic agent** for treatment of overactive bladder or urge incontinence {sensorium impairment}

Section L: Analgesic Drugs**.**

L1. Use of oral or transdermal strong opioid [morphine, oxycodone, fentanyl, buprenorphine, diamorphine, methadone, tramadol, pethidine, pentazocine] as first line therapy for mild pain (WHO analgesic ladder not observed; *paracetamol or NSAID not prescribed as first-line therapy*).

∆ L2. Opioid in *daily* regular use (as distinct from PRN) without concomitant laxative {severe constipation}

∆ L3. Long-acting opioid without short-acting opioid for break-through *moderate or severe* pain {severe pain}

**L4. Lidocaine** (lignocaine) patch for chronic osteoarthritis pain (no clear-cut evidence of efficacy).

**L5. Gabapentinoid** [e.g., gabapentin, pregabalin] for non-neuropathic pain (lack of evidence of efficacy).

**L6. Paracetamol** ≥ 3 g/24 hours with poor nutritional status [BMI < 18 kg<<km²] or chronic liver disease {hepatotoxicity}

Section M: Antimuscarinic/anticholinergic drug burden.

M1: Concomitant use of ≥ 2 drugs with antimuscarinic/anticholinergic properties [e.g. bladder antispasmodics, intestinal antispasmodics, tricyclic antidepressants, first generation antihistamines, *antipsychotics*] {increased anticholinergic toxicity}

Appendix 2b. START.v3 criteria.

New criteria in bold. Criteria with significant *modifications* are pointed by *∆* (modifications in italic). ~~Removed~~ in SS.v3

**Section A**: **Indicated drugs**

**A1. Where a drug is clearly indicated and considered appropriate** in the particular clinical context and there is no clear contraindication, that drug should be initiated as per formulary guidelines for dose and duration.

Section B: Cardiovascular System

∆ B1. Antihypertensive therapy for systolic blood pressure ~~(> 160)~~ *> 140 mmHg* and/or diastolic blood pressure > 90 mmHg, *unless established moderate-severe physical frailty in whom the diagnostic thresholds are 150 mmHg and 90 mmHg.*

∆ B2. Statin therapy for coronary, cerebral or peripheral vascular disease, unless the patient’s status is end-of-life *or established moderate-severe frailty.*

B3. ACEI (angiotensin converting enzyme inhibitor) for coronary artery disease.

B4. Beta-blocker for *symptomatic (ischaemic)* coronary artery disease.

∆ B5. ACEI (angiotensin converting enzyme inhibitor) for HFrEF (heart failure with *reduced ejection fraction*).

B6. *Cardioselective* beta-blocker (bisoprolol, nebivolol, metoprolol or carvedilol) for stable HFrEF.

**B7. Mineralocorticoid receptor antagonist** [i.e. spironolactone, eplerenone] for heart failure without severe renal function impairment [i.e. eGFR > 30 ml/min/m^2^].

**B8. SGLT-2 inhibitor** [i.e. canagliflozin, dapagliflozin, empagliflozin, ertugliflozin] for symptomatic heart failure, with or without reduced ejection fraction, regardless of diabetes being present or not.

**B9: Sacubitril/valsartan** for HFrEF with persistent heart failure symptoms despite optimal dose of ACEI or ARB (angiotensin receptor blocker), sacubitril/valsartan to replace ACEI or ARB).

**B10. Beta-blocker** for chronic atrial fibrillation with uncontrolled heart rate.

**B11. Intravenous iron** for symptomatic HFrEF (heart failure with reduced ejection fraction) and iron deficiency.

**Section C: Coagulation System**

∆ C1. Anticoagulant [i.e. vitamin K antagonists, direct thrombin inhibitors, factor Xa inhibitor] for chronic *or paroxysmal* atrial fibrillation.

C2. Antiplatelet therapy [i.e. aspirin, clopidogrel, prasugrel, ticagrelor] for coronary, cerebral, peripheral vascular disease.

Section D: Central Nervous System.

D1. L-DOPA or a dopamine agonist for idiopathic Parkinson’s disease with disability due to functional impairment.

∆ D2. Non-tricyclic antidepressant drug for *major depression*.

D3. Acetylcholinesterase inhibitor [i.e. donepezil, rivastigmine, galantamine] for mild-moderate Alzheimer’s dementia.

**D4. Rivastigmine** for Lewy body dementia or *Parkinson’s disease dementia*.

∆ D5. SSRI (selective serotonin reuptake inhibitor) [if SSRI contraindicated : SNRI or pregabalin] for persistent severe anxiety that affects independent functioning *and quality of life.*

D6. Dopamine agonist [i.e. ropinirole or pramipexole or rotigotine] for restless legs syndrome, once iron deficiency and severe chronic kidney disease [*i.e. eGFR < 30 ml/min/m^2^*] have been excluded.

**D7. Propranolol** for essential tremor with disability due to functional impairment

**Section E: Renal System**

**E1. 1α-OH-cholecalciferol or calcitriol** supplementation for severe chronic kidney disease [i.e. eGFR < 30 ml/min/m^2^] with hypocalcaemia [i.e. corrected serum calcium < 2.10 mmol/l] and associated secondary hyperparathyroidism.

**E2. Phosphate binder** for severe chronic kidney disease [i.e. eGFR < 30 ml/min/m2) when serum phosphate concentration persistently > 1.76 mmol/l (5.5 mg/dl) despite adherence to renal diet.

**E3. Erythropoietin analogue** for severe chronic kidney disease (i.e. eGFR < 30 ml/min/m^2^) with symptomatic anaemia not attributable to haematinic or iron deficiency, to achieve a haemoglobin concentration of 10.0 to 12.0 g/dl.

**E4: ARB or ACEI** for proteinuria [i.e. urine albumin excretion > 300 mg/24 h] in chronic kidney disease

Section F: Gastrointestinal System.

F1. Proton pump Inhibitor for severe gastro-oesophageal reflux disease or peptic oesophageal stricture.

**F2.** **Proton pump inhibitor** with low-dose aspirin and history of peptic ulcer or reflux oesophagitis.

**F3. Proton pump inhibitor** with NSAID, short-term (< 2 weeks) or longer-term.

F4. Fibre supplements [e.g., bran, ispaghula, methylcellulose, sterculia] for diverticulosis and history of constipation.

**F5. Osmotic laxative** [e.g., lactulose, macrogol, sorbitol] for chronic benign constipation, idiopathic or secondary.

**F6. Probiotic** when antibiotic therapy, except in immunocompromised or severely debilitated patients {Clostridioides difficile}

**F7. Helicobacter pylori (HP) eradication** therapy for HP-associated active peptic ulcer disease.

Section G: Respiratory System.

∆ G1. *LAMA* [e.g. tiotropium*, aclidinium, umeclidinium, glycopyrronium] or LABA* [e.g., bambuterol, formoterol, indacaterol, olodaterol, salmeterol] for symptomatic chronic asthma or COPD GOLD 1-2

∆ G2. Inhaled corticosteroid *in daily use* [e.g., beclomethasone, budesonide, ciclesonide, fluticasone, mometasone] for moderate-severe asthma or COPD of *GOLD 3-4 severity*, where FEV1 <50% of predicted value and repeated exacerbations requiring treatment with oral corticosteroids.

G3. Home continuous oxygen for chronic hypoxaemia (i.e. pO2 < 8.0 kPa or 60 mmHg) or SaO2 ≤ 88 %.

Section H: Musculoskeletal System.

H1. DMARD (disease-modifying anti-rheumatic drug) for *chronic,* active and disabling rheumatoid arthritis.

H2. Bisphosphonate + vitamin D + calcium for long-term systemic corticosteroid therapy {prevention of osteoporosis}.

∆ H3. Vitamin D ~~and calcium supplement~~ for osteoporosis [i.e. Bone Mineral Density T-scores below -2.5] and/or fragility bone fracture(s).

∆ H4. Bone anti-resorptive or anabolic therapy [e.g. bisphosphonate, ~~strontium ranelate,~~ teriparatide, denosumab) for osteoporosis (bone mineral density T-scores below -2.5 in one or multiple site) and/or previous fragility fracture(s) in the absence of pharmacological or clinical (life expectancy < 1 year) contraindication.

∆ H5. Vitamin D supplement for *confirmed vitamin D deficiency (25-hydroxycolecalciferol < 20 micrograms/L, < 50 nmol/L)* when housebound or experiencing falls or having osteopenia (-2.5 < bone mineral density T-score < -1.0).

**H6. Anti-resorptive treatment** after discontinuation of ≥ 2 doses of denosumab {rebound increased bone turnover markers, BMD loss, and increased risk of vertebral fracture following denosumab discontinuation}.

**H7. Anti-resorptive treatment** after discontinuation of teriparatide/abaloparatide treatment for osteoporosis.

H8. Xanthine-oxidase inhibitor [e.g. allopurinol, febuxostat] for recurrent episodes of gout.

H9. Folic acid supplement when methotrexate.

Section I: Urogenital System.

∆ I1. *Selective* alpha-1 receptor blocker [e.g., tamsulosin, silodosin] *for lower urinary tract symptoms related to benign prostatic hyperplasia* where prostatectomy is not considered necessary *or appropriate or safe.*

∆ I2. 5-alpha reductase inhibitor *[e.g. finasteride, dutasteride]* for *lower urinary tract symptoms related to benign prostatic hyperplasia* where prostatectomy is not considered necessary *or appropriate or safe.*

I3. Oestrogen, local [topic or pessary] for symptomatic atrophic vaginitis.

**I4. Oestrogen, local** [topic or pessary] for recurrent urinary tract infections in women

**I5. Phosphodiesterase type-5 inhibitor** [e.g. avanafil, sildenafil, tadalafil, vardenafil] for persistent erectile dysfunction that causes distress.

Section J: Endocrine System.

∆ J1. ACEI [or ARB if ACEI intolerance] for diabetes with proteinuria [i.e. dipstick proteinuria or microalbuminuria (> 30 mg/24 hours)] *unless severe CKD (eGFR < 30 ml/min/m^2^)*

Section K: Analgesics criteria.

∆ K1. High-potency opioids in moderate-severe *non-arthritis* pain, where paracetamol, NSAIDs or low-potency opioids are not appropriate to the pain severity or have been ineffective.

∆ K2. Laxatives with opioids regularly used *[i.e. other than PRN use*]

**K3. Topical 5% lidocaine** [lignocaine] patch for localized neuropathic pain [e.g. post-herpetic neuralgia**]**

Section L: Vaccines criteria.

∆ L1: Seasonal *trivalent* influenza vaccine annually.

L2: Pneumococcal vaccine at least once according to national guidelines.

**L3. Varicella-zoster vaccine** according to national guidelines.

**L4. SARS-CoV2 vaccine** according to national guidelines.

Abbreviations : ACEI: Angiotensin-converting-enzyme inhibitors, ARB: angiotensin II receptor blocker, COPD: chronic obstructive pulmonary disease, COX-2: Cyclooxygenase-2, dBP: diastolic blood pressure, eGFR: estimated glomerular filtration rate, HFpEF : heart failure with preserved ejection fraction, HFrEF : heart failure with reduced ejection fraction, LABA: long-acting beta-agonists, LAMA: long-acting muscarinic antagonists, NSAID: nonsteroidal anti-inflammatory drugs, NYHA: New York Heart Association, pCO2: partial pressure of carbon dioxide, pO2: partial pressure of oxygen, PPI: proton pump inhibitor, PRN : pro re nata, SARS-CoV2: Severe acute respiratory syndrome coronavirus 2, sBP: systolic blood pressure, SGLT2: sodium-glucose cotransporter 2, SGLT2: sodium-glucose cotransporter 2, SNRI: Serotonin–norepinephrine reuptake inhibitors, SSRI: Selective Serotonin Reuptake Inhibitor, T4: Thyroxine, TSH: thyroid stimulating hormone, WHO : World Health Organization
